# Service evaluation of weight gain in patients prescribed antipsychotics within the early intervention service

**DOI:** 10.1192/bjo.2021.327

**Published:** 2021-06-18

**Authors:** Louisa Ward, Charlotte Marriott, Godwin Tong

**Affiliations:** worcestershire health and care trust

## Abstract

**Aims:**

To assess physical health in patients under the Early Intervention Service, whom are prescribed antipsychotics.

To consider whether further intervention needs to be provided or promoted to improve physical health in this group.

**Method:**

Assessment of carenotes database for all 63 patients on EIS caseload prescribed antipsychotics.

**Result:**

Out of 47 patients studied, 20 were non-smokers at baseline. 25% of them ended up becoming smokers by the end of the study time.

Out of 47 patients studied 28 were non-drinkers at baseline. 32% of them ended up engaging in alcohol by the end of the study time.

Out of 47 patients studied, 38 patients had data available to record weight changes per year. Out of the 38 patients, 27 of them had positive weight change; average weight change was + 6.38 kg per year. The highest weight gain was 38.4 kg, the highest weight lost was 47.3 kg.

Out of 47 patients studied, 35 patients had data available to record BMI changes. Out of the 35 patients, 27 of them had positive BMI increases, average BMI change was + 2.68. The highest BMI increase was 12.84. The highest BMI decrease was 8.24.

Out of 47 patients studied, 11 patients had data available to record random glucose level changes. Out of the 11 patients, 7 of them had increased glucose levels, average glucose change were + 0.5mmol/l. The highest increase in glucose was 3.9mmol/l and the highest drop in glucose was 2.6mmol/l.

Out of 47 patients studied, 19 patients had data available to record HbA1c levels. Out of the 19 patients, 10 of them had increased HbA1c levels, with the average change being + 0.31 mmol/mol. The highest increase in HbA1c levels was 5 mmol/mol and the highest drop in HbA1c levels was 3 mmol/mol.

Out of 47 patients studied, 30 patients had data available to record cholesterol changes. Out of the 30 patients, 21 of them had increased cholesterol levels, with the average change being + 0.09mmol/l. The highest increase in cholesterol was 1.7mmol/l and the highest drop in cholesterol levels was 2.6.

Taken together, we show that anti-psychotic use has a negative effect on physical health parameters such as weight gain, BMI increase, HbA1c levels and cholesterol levels. This increases the patient's risk of developing diabetes/metabolic syndrome in the future.

**Conclusion:**

Re-audit.

